# Dentin wettability enhancement for three irrigating solutions and their effect on push out bond strength of gutta percha / AH Plus

**DOI:** 10.4317/jced.51865

**Published:** 2015-04-01

**Authors:** Sarah-Hossam Fahmy, Abeer-Abdel-Hakim El Gendy, Salma-Hassan El Ashry

**Affiliations:** 1 Assistant Lecturer at the Endodontic Department, Faculty of Dentistry, Ain Shams University; 2 Associate Professor at the Endodontic department, faculty of Dentistry, Ain Shams University; 3 Professor at the Endodontic department, faculty of Dentistry, Ain Shams University

## Abstract

**Background:**

The aim of this study was to investigate the effect of wettability enhancement for 17% EDTA, 2.5% sodium hypochlorite and 7% maleic acid solutions on push out bond strength of gutta percha /AH Plus to root dentin.

**Material and Methods:**

One hundred and eight extracted single rooted human lower premolars were instrumented up to Protaper Universal F5 then irrigated with 3ml of 2.5% NaOCl after each file. Irrigants were prepared and a pilot study for determination of Tween 80 concentration yielding the lowest surface tension value in every solution was conducted. Samples were randomly divided into a control group and two experimental groups (17% EDTA and 7% Maleate), further split into eight subgroups (n=12), according to Tween 80 implementation sequence. Roots were obturated using gutta percha and AH plus by lateral condensation. Bond strength was measured by push out test. Mode of failure was then evaluated quantitatively by stereomicroscopy. Data were statistically analyzed using one way ANOVA followed by Tukey-Kramer for multiple comparisons.

**Results:**

Control group showed the lowest values. Maleic acid subgroups showed significantly higher overall values than EDTA subgroups (P<0.05). Protocols implementing surfactant containing NaOCl showed significantly lower values than plain counterparts. Failure pattern was predominantly cohesive for plain regimens and the ones implementing Tween 80 in maleic acid solutions with plain NaOCl.

**Conclusions:**

Tween 80 addition to demineralizing irrigants increased the bond strength values. Surfactant containing NaOCl solutions yielded lower bond strength than plain ones.

** Key words:**Wettability enhancement for three irrigants vs. corresponding gutta percha/AH Plus bonding.

## Introduction

Interfacial adaptation between root canal filling materials and dentinal walls is a pivotal issue for long term success of endodontic treatment. This multifactorial issue relies on irrigants influence on dentinal substrate physically and chemically besides the obturation efficiency (1).

Several chemical agents are employed for debridement and disinfection of root canals. Sodium hypochlorite is commonly used for removal of organic components whereas demineralizing agents like EDTA (ethylene diamine tetraacetic acid) and maleic acid are targeted towards the inorganic portions. The application of these conditioning agents to the dentinal substrate modifies its physicochemical properties as well as the proportions of its organic and inorganic phases (2,3).

Addition of surfactants reduces fluids surface tension which in turn would enhance their wettability properties (4). Surfactants exist in three categories anionic, cationic or non-ionic. Tween 80 is a non-ionic tenside, approved by the FDA with a wide spec-trum of medical applications. It improved the chelation potential and demineralization kinetics of 4.5% citric acid in MTAD solution at a volume of 0.5% (5).

A better contact of the irrigants with canal walls would be clinically implied in cleanliness enhancement and better interfacial proximity between fillings and dentin, thereby increasing its strength and fracture toughness (6-8). Different obturation materials require different dentin pretreatments for optimal bonding. Among the sealers used AH Plus showed high levels of biological and physical performances. It has good sealability and resistance to dislodgement (6,7).

Literature showed controversial outcomes with different implementation attempts of surfactants (4-6,8,9), irrigants and irrigation protocols (10-14). Chemically different irrigants with different sequences of application were studied adding up to the variability of the outcomes of bonding quality with different obturation materials (1,6-8,11-14).

The different approaches for surfactants implementation relied upon readily incorporated tensides where the rationale for concentration selection was not disclosed, and the extent of suitability of the surfactant to the main reagent varied with the different chemical combinations for both of them (5,6,8,9). Bukiet *et al.* (4) addressed the issue of precise selections for the concentrations optimally enhancing the wettability properties but without correlating them to the bonding issue. The tenside value optimally reducing surface tension is known as the critical micellar concentration (cmc), chemically corresponding to the point of surfactant molecules aggregation into micelles. The purpose of this study was to assess the bond strength of the GP/AH Plus after using irrigation regimens based on adding Tween 80 to 2.5% NaOCl, 7% maleic acid and 17% EDTA solutions at the cmc value.

Keywords: AH Plus, bond strength, EDTA, maleic acid and Tween 80.

## Material and Methods

-Preparation of Samples

One hundred and eight recently extracted single canaled human lower premolars were selected. They were cleaned using ultrasonic scalers then immersed in 5.26% sodium hypochlorite solution for 30 minutes then decoronated to 16 mm long root segments.

Patency of the root canals was ensured using #10 K-file (Mani inc, Tochigi, Japan). Working length was established 1 mm short of the apex. Apices were blocked with increments of molten green stick compound (Kerr Corporation, CA, USA) (14).

Canals were instrumented with protaper universal rotary files (Dentsply Tulsa Dental Systems, Johnson City, TN) in a crown down fashion till size F5 and irrigated with 3 ml of 2.5% NaOCl at each file change (15).

-Preparation of Final Flush Solutions 

All solutions were prepared at room temperature. PH values were measured by a laboratory PH meter (Mettler Toledo International, Greinfensee, Switzerland) before and after surfactant mixture implementation.

2.5% w/v NaOCl: Equal volumes of 5% sodium hypochlorite (Alexandria for detergents and chemicals, Alexandria, Egypt) and distilled water were mixed. The pH value for plain solution was 12.13 (16).

17 % w/v EDTA and 7 % w/v Maleic Acid: Seventeen gms of disodium edtate salt and Seven gms of maleic acid anhydride respectively (Alexandria for detergents and chemicals, Alexandria, Egypt) were dissolved in 100 mL of distilled water. 5 gms of NaOH crystals were added to the edtate salt to facilitate its dissolution. PH values were 9.2 for EDTA and 2.26 for maleic acid (16).

-Surfactant Addition

Tween 80 was combined with 70% Ethanol co-surfactant (INTERNATIONAL COMPANY for sup. and med. industries, Giza, Egypt) in a ratio of 1:1 as adjuvant co surfactant enhancing the hydrophilicity of the surfactant mixture (17). A pilot study was conducted to determine the Tween 80 critical micellar concentration producing the lowest surface tension and contact angle values for every irrigant. The range of implemented values was between 0.1 till 1.5 ml by volume from the main solution.

**Figure 1:**
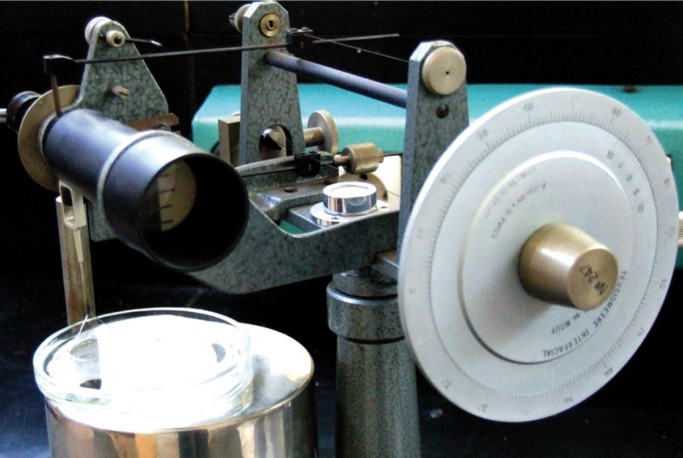
Du Nuoy ring tensiometer for interfacial surface tension measurement.

Interfacial surface tension was measured using the Du Nuoy ring tensiometer, (CSC Scientific company inc, VA, USA), (Fig. 1) (18). The functionality of this tensiometer was based on the gradual insertion of a platinum iridium ring in the fluid to be tested, then its gradual withdrawal till the formed fluid lamella in the ring gets detached.

The interfacial tension is the maximum force needed to detach the ring to pull it free from the aqueous liquid surface. Five readings were taken for each solution, and the average was recorded.

Wettability assessment was performed by the sessile drop technique. Five drops were placed on flat dentin disks and 1 minute was allowed for their settlement before capturing images with a computer-controlled display video camera to take pictures. The photos were submitted to a software program, (Image-J v 1.44, U.S. National Institute of Health, Bethesda, Maryland, USA) where the contact angle was then the angle comprised between the baseline (liquid-solid interfacial line) and the profile line (liquid/vapor interfacial line).

Ambient temperature was set at 22˚C considering the influence of temperature change and humidity on surface tension coefficient (4). Optimal surfactant concentration values were 0.6%, 0.9% and 1% for 2.5% NaOCl, 17% EDTA and 7% maleic acid respectively. PH measurements after adding the surfactant mixture were 11 for NaOCl, 6.4 for EDTA and 1.18 for maleic acid.

-Classification of Samples 

Classification was based on the final irrigant employed with NaOCl: Group I (17% EDTA, n=48); Group II (7% Maleic acid, n=48) and a positive control group (distilled water, n=12). Each experimental group was further divided into four subgroups (n=12) according to the presence or absence of Tween 80. Subgroup IA: 17%EDTA solution followed by 2.5% NaOCl solution. Subgroup IB: 17% EDTA solution with added 0.9% Tween 80 followed by 2.5% NaOCl solution. Subgroup IC: 17% EDTA solution followed by 2.5% NaOCl solution with 0.6% Tween 80.Subgroup ID: 17% EDTA solution with 0.9% Tween 80 followed by 2.5%NaOCl with 0.6% Tween 80.

Subgroup IIA: 7% maleic acid solution followed by 2.5% NaOCl solution.

Subgroup IIB: 7% maleic acid solution with 1% Tween 80 followed by 2.5% NaOCl solution. Subgroup IIC: 7% maleic acid solution followed by 2.5% NaOCl solution with 0.6% Tween 80. Subgroup IID: 7% maleic acid solution with1% Tween 80 followed by 2.5% NaOCl solution with 0.6% Tween 80. Control group: Distilled water.

The protocol was based on final flush with five milliliters of the decalcifying agent followed by the same volume of 2.5% NaOCl using stropko NiTi Flexi; (SybronEndo, Orange, CA) Tip to within 1-2 mm from the working length for 1 minute (18).

Five milliliters of distilled water were injected for one minute at the end. Canals were dried with paper points (Diadent Group International inc, Chongju, Korea).

-Push out Bond Strength Determination

Obturation was carried out by lateral condensation of gutta percha and AH Plus (DENTSPLY, Tulsa dental specialities, Oklahoma, USA). The later was mixed according to manufacturer’s instructions. Roots were centrally positioned in a cylindrical mold 1 cm in diameter and embedded vertically in acrylic resin (Acrostone) (19). Three sections of 2 mm±0.1 thickness were cut then confirmed by a digital vernier caliper (Guilin measuring and cutting tools Co, Ltd, Guanxi, China) then scanned for measurement of coronal and apical diameters of the obturated areas by a CanoScan LiDE Scanner (Canon, Tokyo, Japan). The radii were estimated by the NIH Image J software (National Institutes of Health, Maryland, USA).

Each sample was subjected to compressive loading via a computer controlled materials testing machine, (Nexygen-MT, Lloyd Instruments, Fareham, UK) with a load cell of 5kN at a crosshead speed of 0.5 mm/ min. Load was applied by 3 plungers of different tip diameters (1 mm, 0.75 mm & 0.5 mm) in an apico-coronal direction. The selected diameter of the plunger was positioned so that it only contacts the filling. Failure was manifested by extrusion of filling material and confirmed by sudden drop along load-deflection curve as recorded by computer software (Nexygen data-analysis software, Lloyd). Maximum failure load was recorded in N and converted into MPa to determine the push out bond strength using the following formula (6): Push out bond strength (MPa) = F (N)/A (mm2). The adhesion surface area of each section was calculated as.

(πr1 + πr2) x L. L was calculated as √((r1-r2)² )+h2 where π is the constant 3.14, r1 is the smaller radius, r2 is the larger, and h is the thickness of the section in mm. Where F is the maximum load, A is the adhesion area, π is the constant 3.14, r1 apical radius, r2 coronal radius, h is the thickness of the sample in millimeters. 

-Mode of Failure Evaluation

•Stereomicroscopic Evaluation

Sections were imaged by stereomicroscope (Wild M3B; Leica, Heerbrugg, Switzerland) at 40 x magniﬁcation to determine the nature of bond failure.

Debonded specimens were categorized into 1 of 3 failure modes according to Skidmore *et al.* (20) as: Type I (adhesive failure at the sealer-dentin interface), type II (cohesive failure within the sealer or gutta percha) and type III (mixed failure). Data were statistically analyzed using the one way ANOVA followed by post hoc tests for pair wise comparisons. SPSS-17 software was employed.

•Scanning Electron Microscopic Evaluation 

A selective sample of five roots from each group were collected after push out testing and prepared for scanning electron microscopic (SEM) examination (Quanta FEG SEM; FEI Co, Hillsboro, OR). The samples were kept in in 2Molar hydrochloric acid (HCl) for 48 hours to ensure complete dental tissue demineralization (19). Then, the samples were thoroughly rinsed, freeze dried, sputter coated with carbon, for observation of the interfaces including the canal wall and the surface of the debonded root filling material.

## Results

**Figure 2:**
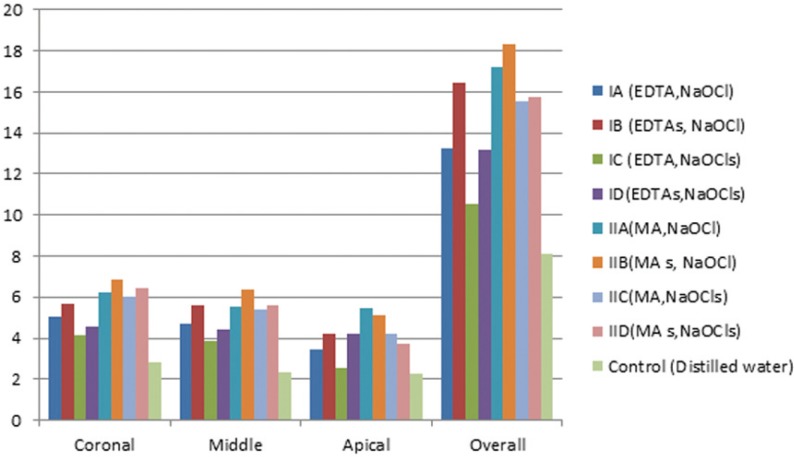
Column chart showing the mean push out bond strength values of gutta percha/AH Plus among the different irrigation protocols at the same root canal level.

**Figure 3:**
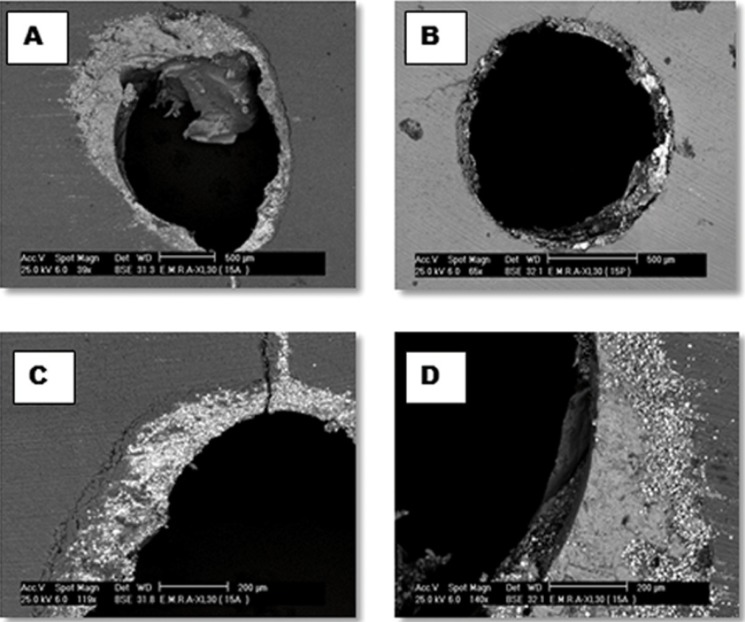
SEM images of the different modes of failure. (A) Cohesive failure. (B) Mixed failure. (C) Higher magnification for (B) showing one side of root canal lumen with adhesive mode of failure at the sealer/ dentin interface, a gritty layer of sealer is left on dentinal wall (D) Higher magnification for (B) showing mixed failure ; adhesive at sealer/gutta percha interface and cohesive within gutta percha respectively.

Figure 2 showed that the control group had the lowest value, (*P*<0.05). Subgroups IIA and IIB had significantly higher overall values than the other subgroups, (*P*<0.05), subgroup IC had the lowest value. Coronally maleic acid subgroups recorded significantly higher values than EDTA subgroups. At the middle level subgroup IIB had significantly the highest value while the lowest was noted with subgroup IC. Apically subgroups IIA and IIB yielded significantly higher values while subgroups IA, IC and IID recorded the lowest values. Comparison between three levels revealed that coronal had higher values than middle and apical. Failure patterns were predominantly cohesive for subgroup IIB and IIA, and purely adhesive for the control group ( Table 1, Fig. 3).

**Table 1:**
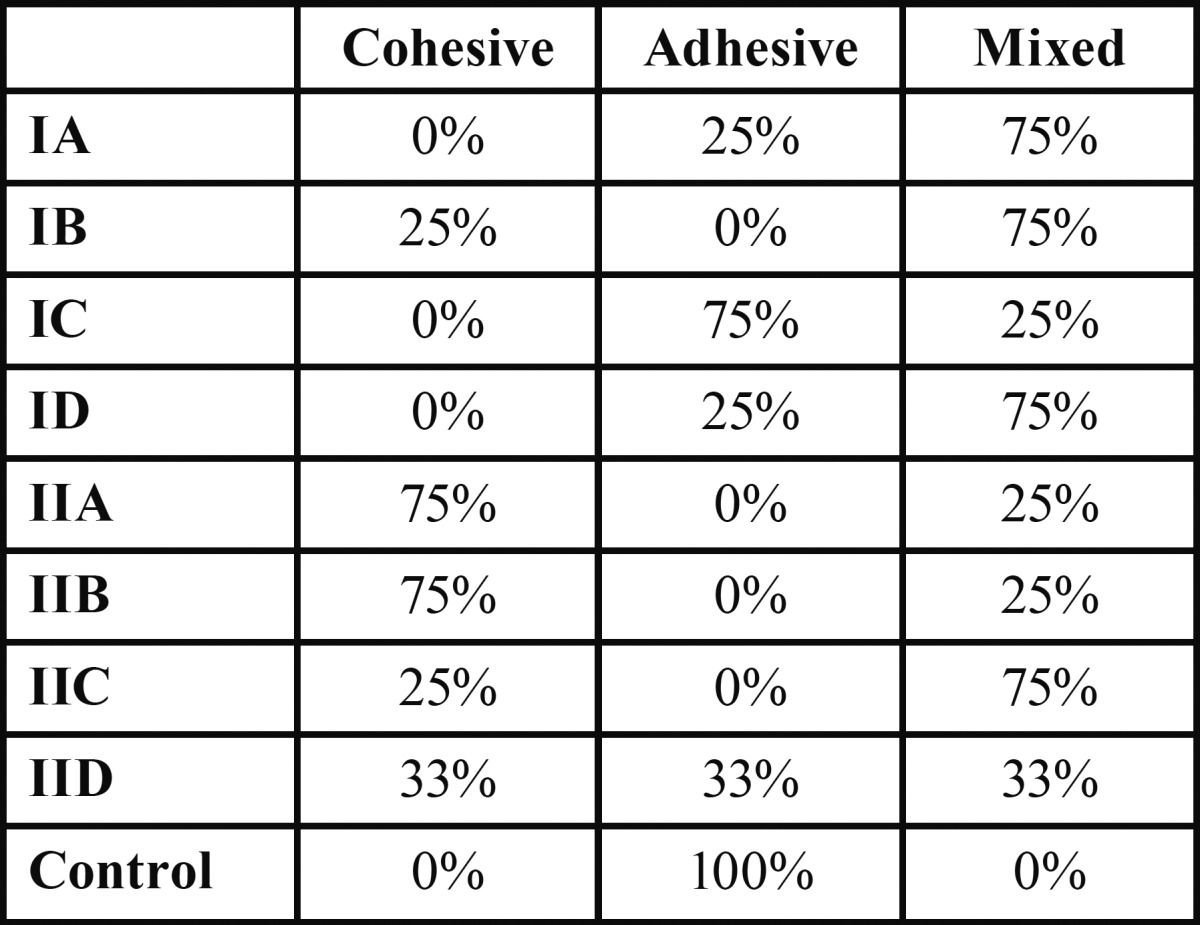
Different modes of failure among the different irrigation protocols.

## Discussion

Root canal irrigation and dentin pretreatment with various protocols affect the surface properties of the dentinal substrate as well as its composition. Surfactants implementation is supposed to improve the permeation and cleaning potentials of the different irrigants. This would influence the adaptation of different root filling /sealers systems (6-8,11-14).

The present study showed that Tween 80 incorporation at the critical micellar concentration (cmc) differentially affected the resultant bond strength values of gutta percha /AH Plus to root dentin. The (cmc) corresponds to the point at which the surfactant aggregates (micelles) start to form in the fluid bulk after the monomeric surfactant molecules saturate the fluid surface (4).

Experimental groups recorded higher bond strength values than the control group where distilled water was used, probably because dentin surface remained covered with debris interfering with resin penetration into dentinal tubules (21). This could also be due to the residual moisture in the debris layer, adversely affecting epoxy resin monomer conversion , leading to incomplete resin polymerization and decreased bond strength to dentin , so the weak link lied at the sealer-dentin interface (22,23). Maleate showed better outcomes than EDTA. Within every group, Tween 80 addition to the demineralizing agent gave the best outcome, followed by the plain regimens, then when added to the demineralizing agent and NaOCl, and finally when implemented in NaOCl only.

The interpretation could be based upon micromechanical and chemical aspects.

Ballal *et al.* (24) postulated that maleate increases tubular patency and surface roughness for its low pH. They attributed it to its higher cleaning and demineralization potentials. Furthermore, in this study Tween 80 as a surfactant remarkably enhanced maleic acid wettability. The interaction resulted from non-polar hydrogen bonding between the carboxylic groups of maleate and the oxygen of the ethylene oxide chain of Tween 80 (Polysorbate 80). Important conformational changes were manifested by the decrease of Tween 80 concentration at the air/water interface. The tenside is adsorbed onto the acidic polymer and drawn into the bulk solution “tight host guest inclusion “ as described by Barreiro-Iglesias *et al.* (25), thereby enhancing its wettability.

Difference among the subgroups is based on influence of pH values of demineralizing agent on NaOCl tissue solvent action (26-30). This highlights the importance of collagen substrate for epoxy resin bonding (14). Tween 80 addition to EDTA and ma-leate reduced their pH values which might have released more free available chlorine in solution in than the plain counterparts. Chlorine gas is volatile and unstable.

The collagenolytic effect of NaOCl is reduced thereby preserving the partially demineralized collagen necessary for AH Plus covalent bonding via the open epoxide rings on surface. Clarkson *et al.* (26) found out that surfactant implementation in NaOCl reduces chlorine loss in comparison to the plain counterparts. Subgroups A and B had plain NaOCl solutions therefore experienced higher chlorine loss than subgroups C and D which had surfactant.

The importance of collagen substrate integrity was also shown by De Assis *et al.* (11) who concluded that NaOCl deproteinizes dentinal substrate. The outcome is a hydrophilic surface that would interfere with the hydrophobic nature of AH Plus. Tuncer *et al.* (7) recorded a higher penetration depth of AH Plus after final irrigation with EDTA, maleic acid and citric acid. Conversely Hashem *et al.* (6) noted that MTAD reduced push out bond strength of gutta percha/AH Plus. They correlated it to Tween 80 mediated enhancement of dentinal permeability and exposure of dentinal fluid interfering with AH Plus hydrophobicity. From another perspective Tay *et al.* (8) reported that AH Plus suboptimally infiltrated the calcium depleted dehydrated collagen matrixError! Reference source not found after final MTAD and EDTA rinses as a consequence of intrafibrillar bonding.

Coronal and middle levels showed higher values than the apical segment which could be linked to better cleanliness. There are more dentinal tubules with larger diameter in the coronal area than in the middle and apical ones.

Failure analysis correlate with the push-out test results in that higher bond strength reduced the likelihood of disruption of the sealer-dentin interface i.e. failure was cohesive within the sealer. This was manifested in the subgroups where Tween 80 was added to the demineralizing agent and with the plain regimens. Subgroups with increased debris fraction manifested a debonding at the sealer-dentine interface.

## Conclusions

Under the conditions of the present study it was concluded that Tween 80 addition to the demineralizing irrigants improved the bond strength value of gutta percha/AH Plus to radicular dentin whereas its addition to NaOCl gave lower results. Further cyto-toxicity study is required to biologically investigate the interaction outcome between Tween 80 and the employed irrigants.
